# Single-Cell RNA Sequencing Reveals the Spatial Heterogeneity and Functional Alteration of Endothelial Cells in Chronic Hepatitis B Infection

**DOI:** 10.3390/ijms25137016

**Published:** 2024-06-27

**Authors:** Jingqi Shi, Qingyu Li, Jian Li, Jianglin Zhou, Xiaochang Zhang, Shengqi Wang, Liang Guo

**Affiliations:** Bioinformatics Center of AMMS, Beijing 100039, China; shijingqi@bmi.ac.cn (J.S.); liqingyu@bmi.ac.cn (Q.L.); jianli522_china@163.com (J.L.); zjianglin@163.com (J.Z.); zhangxc@bmi.ac.cn (X.Z.)

**Keywords:** Hepatitis B, single-cell transcriptomics, endothelial cells, NF-κB signaling pathway, capillarization, angiogenesis, cell–cell interactions

## Abstract

Chronic Hepatitis B virus (CHB) infection is a global health challenge, causing damage ranging from hepatitis to cirrhosis and hepatocellular carcinoma. In our study, single-cell RNA sequencing (scRNA-seq) analysis was performed in livers from mice models with chronic inflammation induced by CHB infection and we found that endothelial cells (ECs) exhibited the largest number of differentially expressed genes (DEGs) among all ten cell types. NF-κB signaling was activated in ECs to induce cell dysfunction and subsequent hepatic inflammation, which might be mediated by the interaction of macrophage-derived and cholangiocyte-derived VISFATIN/Nampt signaling. Moreover, we divided ECs into three subclusters, including periportal ECs (EC_Z1), midzonal ECs (EC_Z2), and pericentral ECs (EC_Z3) according to hepatic zonation. Functional analysis suggested that pericentral ECs and midzonal ECs, instead of periportal ECs, were more vulnerable to HBV infection, as the VISFATIN/Nampt- NF-κB axis was mainly altered in these two subpopulations. Interestingly, pericentral ECs showed increasing communication with macrophages and cholangiocytes via the Nampt-Insr and Nampt-Itga5/Itgb1 axis upon CHB infection, which contribute to angiogenesis and vascular capillarization. Additionally, ECs, especially pericentral ECs, showed a close connection with nature killer (NK) cells and T cells via the Cxcl6-Cxcr6 axis, which is involved in shaping the microenvironment in CHB mice livers. Thus, our study described the heterogeneity and functional alterations of three subclusters in ECs. We revealed the potential role of VISFATIN/Nampt signaling in modulating ECs characteristics and related hepatic inflammation, and EC-derived chemokine Cxcl16 in shaping NK and T cell recruitment, providing key insights into the multifunctionality of ECs in CHB-associated pathologies.

## 1. Introduction

A chronic infection of the Hepatitis B virus (HBV) can result in a high risk of liver cirrhosis and hepatocellular carcinoma which causes high morbidity and mortality, and is a global threat to public health [1,2]. However, immune surveillance of the host during HBV-associated pathogens is a challenge that remains to be resolved. Therefore, there is an urgent need to reveal the mechanisms of the complicated viral–host interactions and lay a foundation for novel therapeutic strategies to achieve a functional cure for HBV.

It is well known that HBV could induce a more immunosuppressive and exhausted environment in hepatocarcinogenesis. HBV infection induces PD-1^hi^ atypical memory B cells, impairing antibody production and B cell immunity [3]. Neutrophils from HBV-related acute-on-chronic liver failure (HBV-ACLF) patients displayed impaired phagocytosis [4]. Immune-metabolism disorder plays an indispensable role in HBV-ACLF progression [5]. Kupffer cells of offsprings displayed a highly PD-L1 expressing, immune-suppressive phenotype, owing to maternal HBeAg [6]. Tumor-associated Mφ in HBV-related HCC suppressed T cell infiltration [7]. Moreover, regulatory T cells and CD8+ resident memory T cells from HBV-associated HCC exhibited high expression of PD-1, with upregulation of KLRC1(exhaustion marker) and downregulation of FCGR3A (activation marker) in NK cells [7,8]. Moreover, HBsAg-specific CD8+ T cells were reported to be a critical trigger to HCC tumorigenesis [9]. A study showed that most of the infused virus homed to the endothelium and ~10% went to macrophages in the liver [10]. Hence, the role of endothelial cells in viral infection cannot be underestimated. As important scavengers in the body and playing critical roles in immune surveillance against intrahepatic pathogens, liver sinusoidal endothelial cells (LSECs) and vascular ECs, which are sensitive to liver injury, have been well known for many years [10,11,12]. For instance, liver injury impairs the cellular function of LSECs to handle oxidative stress mediated by the selective loss of autophagy, which protects LSECs from oxidative stress and maintains LSECs homeostasis, finally aggravating fibrosis progression [13]. In the liver fibrotic process, LSECs usually showed dysregulation phenotypes [12], including capillarization, and lack of LSEC fenestration, etc. LSECs can also modulate the activation of hepatic immune cells during hepatic pathology [14]. The antigen-presenting ability of LSECs was reported to play roles in enhancing T cell responses and inhibiting HBV replication [15]. A recent study underlined that HBeAg produces increasing LSEC-derived TNF-α and IL-27 to trigger HBV-specific T cell activation [16]. However, endothelial dysfunction and capillarization lead to liver inflammation through interfering immune homeostasis [17]. ECs also play a role in driving progression of CHB-associated pathologies. Preety et al. [18] revealed that EC-derived TGF-β may enhance CD133, a stemness marker, to trigger epithelial–mesenchymal transition in HBV-induced HCC.

Moreover, the blood flows in each liver lobule create graded microenvironments with gradients of oxygen and nutrients. Not surprisingly, hepatic cells such as hepatocytes, ECs, and HSCs have zone-specific phenotypes and roles in the liver [19,20,21,22,23]. The emergence of single-cell RNA sequencing (scRNA-seq) technology has helped us to recognize the importance of cellular zone-specific function. A study identified various subpopulations of ECs, including clusters of LSECs and vascular ECs by generating transcriptomic profiles of ECs extracted from human livers [22]. Halpern et al. [24] revealed the zonation patterns of liver ECs in mice by combining the spatial distribution and landmark genes of hepatocytes and adjacent ECs using paired-cell RNA-seq. Su et al. [23] isolated liver ECs from EC-specific green fluorescent protein (GFP) reporter mice and identified heterogeneous EC subpopulations to study phenotypic alterations of ECs in cirrhotic liver. Thus, characterizing ECs according to the zonal distribution is key to understanding their functions in liver physiology and pathophysiology. It is reported that capillarization is most severe in pericentral LSECs [23], indicating its vulnerability, which is attributed to the decreasing concentration of oxygen and nutrition along the direction of blood flow in liver lobules. Chen et al. [25] and Winkler et al. [26] revealed that *Delta-like ligand 4 (DLL4)* and genetic *Gata4* deficiency mediates LSEC capillarization and liver fibrogenesis, respectively. The hepatocytes–LSECs interaction in the pericentral region may aggravate this type of capillarization, which may be related to VEGF-related signaling in cirrhotic livers, with low expression of VEGF receptor *Kdr* and *Nrp1* in pericentral ECs [23,27].

However, the cellular and molecular mechanisms of ECs dysfunction and capillarization during CHB infection remain key unanswered questions. Fortunately, scRNA-seq transcriptome facilitates us to explore the subdivisional function of various cell types and cell communications. Here, we generated a profile of hepatic inflammation in CHB mice constructed by intravenous injection with the AAV-mediated HBV gene and generated an adeno-associated virus (AAV)-HBV mice model, an appropriate model for investigating the mechanisms of CHB infection. Then, ECs functional alterations were systematically described, combining the information of zonal heterogeneity and cell interactions. Briefly, we demonstrate that macrophage-derived VISFATIN/Nampt signaling triggers the dysfunction and capillarization of pericentral ECs, as well as a role of pericentral ECs in recruiting NK and T cells via the Cxcl16-Cxcr6 axis, giving a new insight into elucidating the formation of a proinflammatory microenvironment from a cellular perspective during CHB infection.

## 2. Results

### 2.1. Single-Cell Transcriptomic Analysis of Liver Non-Parenchymal Cells (NPCs) upon CHB Infection

We designed a comprehensive profile of chronic liver inflammation from healthy and HBV-infected mice models by using a 10x genomic single-cell sequencing method.

**Figure 1:**
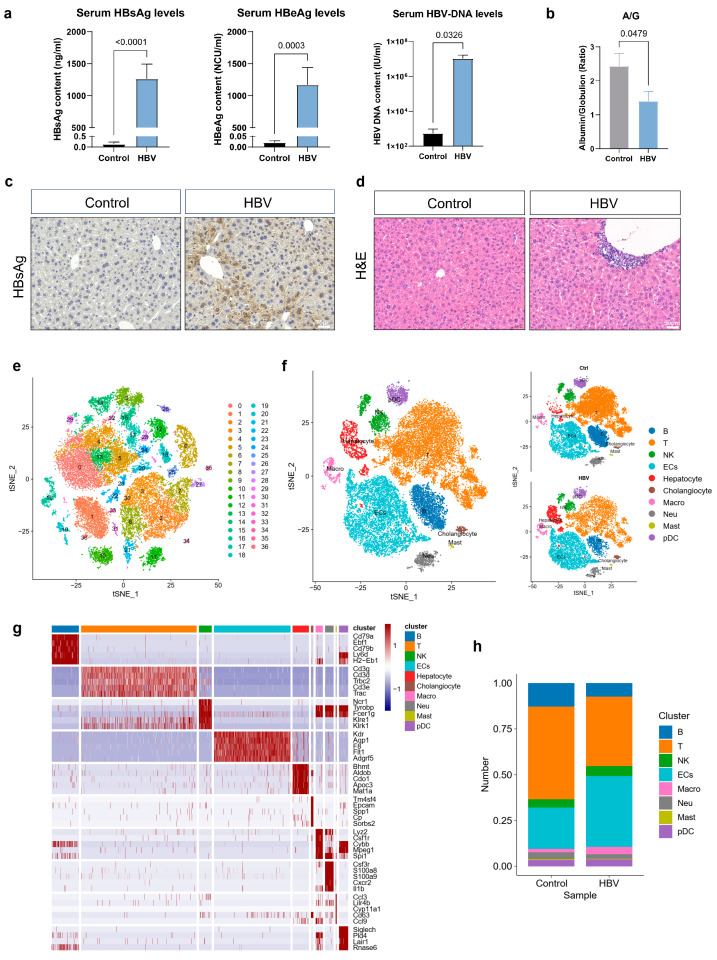
scRNA-seq transcriptomic analysis of cell populations in the CHB liver microenvironment. (**a**) High level of serum HBsAg, HBeAg, and HBV DNA in the HBV mice model (control, N = 3; HBV, N = 5). (**b**) The concentration of the plasma Albumin/Globulin (A/G) ratio in the control (N = 3) and HBV (N = 3) mice model was significantly decreased which was detected by the automatic biochemical analyzer. (**c**) Representative image of the HBsAg-positive area in the liver sections from HBV mice. (**d**) Representative image of liver inflammation in the liver sections from HBV mice. (**e**) t-SNE plot of scRNA-seq data showing 36 cell clusters from control (N = 3) and HBV (N = 3) mice liver. (**f**) Ten cell types were identified. (**g**) Heatmap plot of the top five differentially expressed genes of each cell type. (**h**) The proportion of ECs and macrophages were increased in HBV mice, while the proportion of B cells, T cells and neutrophils were higher in healthy mice.

AAV-HBV mice models [28] induce persistent infection, serving as an appropriate model for investigating the mechanisms of chronic HBV infection. AAV8 infects the liver preferentially, making it a good media to transfer the HBV genome to the mice model. Hence, we generated mice models with CHB infection by intravenously infecting with AAV8 capsids carrying a 1.3-genome length copy of the D genotype HBV genome. After the injection, the serum levels of HBV DNA, HBsAg and HBeAg (Figure 1a) were regularly measured. HBsAg and HBeAg expression, and HBV DNA could be detected in the serum from the HBV mice model but not in those from the healthy mice model. The HBsAg were at about 1000–1500 ng/mL at 6 months post-injection (mpi). The HBeAg and HBV DNA were at 1000–2000 NCU/mL and 1 × 10^7^–1 × 10^8^ IU/mL, respectively. Moreover, the Albumin/Globulin (A/G) ratio were downregulated after CHB infection (Figure 1b). HBsAg-positive cells were detected as being distributed around the liver section by IHC staining (Figure 1c). H&E staining showed immune aggregation and liver inflammation of the mice model at 6 mpi (Figure 1d). Therefore, these results indicated that the HBV models, which simulate CHB development in the liver, were successfully built with high levels of HBV-related indexes.

To investigate cellular heterogeneity and molecular moderation in liver tissue, single-cell RNA analysis was used to portray the profiling of NPCs in the HBV mice model at 6 mpi. We used a total of six livers of the control mice (N = 3) and HBV mice model (N = 3) for library preparation and sequencing. After filtering and normalization of raw expression data, 36 clusters were identified (Figure 1e). Next, the cell cluster annotation was based on known marker genes and ten clusters (Figure 1f) were identified. The expression of representative cell markers are shown by a heatmap plot (Figure 1g), confirming our accuracy of the cluster identity. Further, we found B cell, T cell and neutrophil proportions were decreased after HBV infection. However, cell proportions of ECs and macrophages were increased (Figure 1h).

### 2.2. ECs Exhibited Disturbance in the NF-κB Signaling Pathway

**Figure 2:**
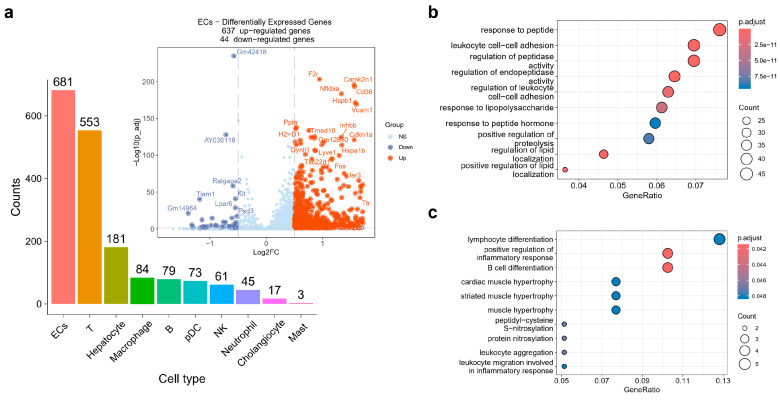

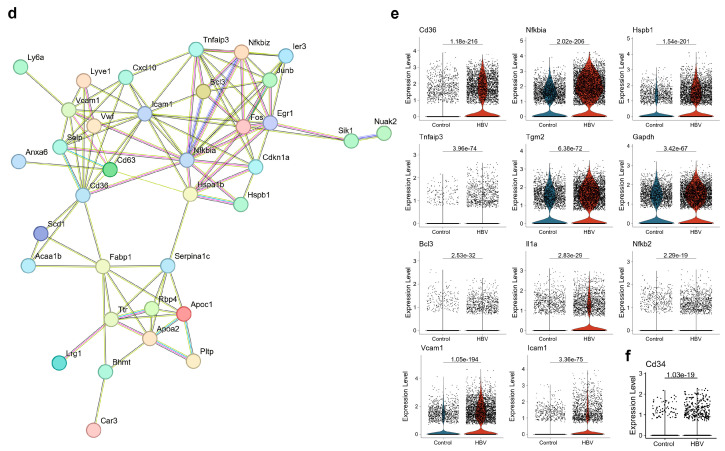
Functional analysis of ECs between healthy and HBV mice models. (**a**) Bar plot shows the DEG counts of each cell types. Volcano plot shows ECs DEGs and marked genes with high log2(fold-change) and low *p*-value. (**b**,**c**) Functional alteration of ECs via GO enrichment analysis using upregulated (**b**) and downregulated DEGs (**c**). (**d**) The protein–protein interaction (PPI) of the intersect genes with top200 log2(fold-change) and top200 statistical significance of upregulated ECs DEGs. (**e**) Vlnplot of NF-κB-associated genes upregulated in HBV mice models. (**f**) Cd34 expression level of ECs was significantly higher in HBV mice models than in control mice models.

To understand the functional alterations, DEGs of each cell cluster were extracted and we found ECs, T cells and hepatocytes, which exhibited the largest number of DEGs among all ten cell types, and these were selected as the top three cell clusters on account of 681 DEGs in ECs, 553 DEGs in T cells, and 181 DEGs in hepatocytes (Figure 2a). The volcano plot of DEGs in ECs between the HBV group and control group is shown in Figure 2a, including 637 upregulated genes and 44 downregulated genes. Next, the GO analysis showed that the upregulated genes in the HBV group were enriched in a set of pathways, including response to peptide, leukocyte cell–cell adhesion, regulation of peptidase activity, regulation of endopeptidase activity and some pathways associated with lipopolysaccharide and lipid localization (Figure 2b). The downregulated genes were enriched in pathways such as lymphocyte differentiation, muscle hypertrophy, protein nitrosylation, and leukocyte aggregation (Figure 2c).

Moreover, the intersect genes with top200 log2(fold-change) and top200 statistical significance of the upregulated DEGs between healthy and HBV mice in ECs were served as input of the protein–protein interaction (PPI) by the STRING database (Figure 2d,e). The results showed that *Nfkbia*, *Icam1*, *Bcl3*, *Nfkbiz*, *Tnfaip3*, *Hspb1*, and *Cd36* were the hub genes in the PPI network, especially *Nfkbia* and *Icam1*. All these genes mentioned above are associated with the NF-κB signaling pathway, indicating that CHB infection promotes NF-κB-mediated inflammation in ECs. Further, the expression level of *Cd34*, a marker of capillarization, was significantly higher in HBV mice models than in control mice models (Figure 2f).

### 2.3. Spatial Heterogeneity of Liver ECs upon CHB Infection

**Figure 3:**
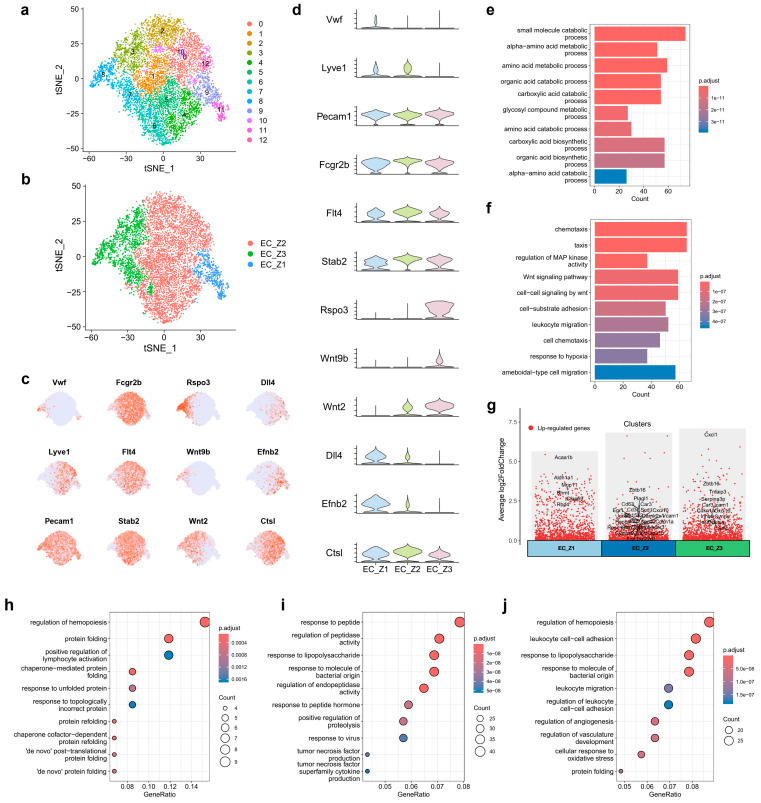
Spatial heterogeneity of ECs upon CHB infection. (**a**,**b**) t-SNE plot showed the ECs subpopulations before (**a**) and after (**b**) annotation. (**c**,**d**) t-SNE plot (**c**) and violin plot (**d**) showed the representative ECs marker gene expression. (**e**,**f**) GO enrichment analysis was used to distinguish the function between periportal ECs (EC_Z1, (**e**)) and pericentral ECs (EC_Z3, (**f**)). (**g**) Manhattan plot visualized the DEGs between healthy and HBV mice in EC_Z1, midzonal ECs (EC_Z2), and EC_Z3, highlighting the intersect genes with top150 log2(fold-change) and top150 statistical significance. (**h**,**j**) GO enrichment analysis showed the upregulated pathways in EC_Z1 (**h**), EC_Z2 (**i**), and EC_Z3 (**j**).

To characterizing ECs according to zonal distribution, 3615 ECs from control mice and 4904 ECs from HBV mice were extracted for further analysis and a total of 12 EC clusters were identified (Figure 3a). It was reported that *Dll4* and *Efnb2* were landmarks of periportal ECs [24] and arterial ECs [29]. *Rspo3*, *Wnt9b*, and *Wnt2* were reported to be enriched in and landmarks of pericentral ECs [24,30]. *Lyve1* and *Ctsl* were landmarks of midzonal ECs [22,31]. In our study, endothelial cells with enriched expression of *von Willebrand factor (Vwf)* are venous ECs, including portal venous ECs (EC1, cluster 11) and central venous ECs (EC5, cluster 8) (Figure S1a). Other endothelial cells with high expression of *Dll4*, and *Efnb2*, limited expression of *Fcgr2b* (*Cd32b*), *Flt4*, *Stab2*, and little expression of *Rspo3*, *Wnt9b*, and *Wnt2* are likely periportal (Zone 1) LSECs (cluster 9). Those endothelial cells with specifically expressed *Rspo3*, *Wnt9b*, and *Wnt2* and little expression of *Dll4* and *Efnb2* were proposed to be pericentral (Zone 3) LSECs (cluster 3, 7). Midzonal (Zone 2) LSECs displayed enriched expression of *Lyve1*, *Fcgr2b*, *Flt4*, *Stab2*, and *Ctsl* (cluster 0, 1, 2, 4, 5, 6, 10, 12). Additionally, all ECs expressed *Cd31* (*Pecam1*), a marker of capillarization and vascular ECs [25,32], with a slightly higher expression in portal venous and central venous ECs. Lymphatic ECs were not found in our data based on its marker genes (Figure S1b).

Collectively, the expression patterns of these representative marker genes (Figure S1c) in our scRNA-seq data led us to define cluster 11 as EC1 (portal venous ECs), cluster 9 as EC2 (zone1, periportal LSECs), cluster 0, 1, 2, 4, 5, 6, 10, 12 as EC3 (zone 2, midzonal LSECs), cluster 3, 7as EC4 (zone 3, pericentral LSECs), and cluster 8 as EC5 (central venous ECs), corresponding to liver zonation (Figure S1a).

After that, GO analysis was used to identify the functional alteration of the 5 clusters upon HBV infection. Interestingly, the functional alteration of EC4 and EC5 upon HBV infection were quite similar (Figure S1d,e). Hence, we believed that EC4 and EC5 were similar in response to HBV infection. Further, EC1 only had nine significantly expressed genes, which was not enough to evaluate its function. As a result, we divided all endothelial cells based on spatial location, combining EC1 and EC2 into EC_Z1 (periportal ECs), EC3 into EC_Z2 (midzonal ECs), and EC4 and EC5 into EC_Z3 (pericentral ECs) (Figure 3b–d).

GO analysis showed the functional characteristics and distinct signaling pathways in periportal and pericentral ECs. Periportal ECs played an important part in the amino acid metabolic process, carboxylic acid catabolic process, and glycosyl compound metabolic process (Figure 3e). Pericentral ECs (Figure 3f) undertook functions associated with cell chemotaxis and migration, MAP kinase activity, Wnt signaling pathway, and cell-substrate adhesion, in line with previous studies [23].

**Figure 4:**
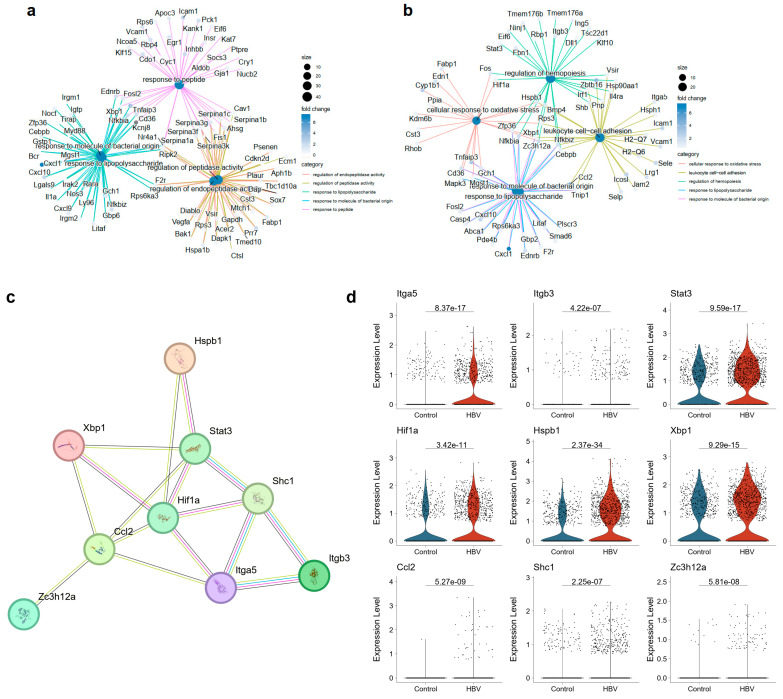
Gene expression characteristics of pericentral ECs. (**a**,**b**) Network showed the correspondence between the top five enriched GO terms and DEGs in EC_Z2 (**a**) and EC_Z3 (**b**). (**c**) PPI network of angiogenesis-associated genes upregulated in HBV mice models. (**d**) Vlnplot of angiogenesis-associated genes shown in the PPI network.

We subsequently identified the functional patterns of these three subtypes of ECs (Figure 3g). Functional alterations of cluster EC_Z1 in CHB infection were mostly enriched in pathways associated with protein folding, such as chaperone-mediated protein folding, response to unfolded protein and topologically incorrect protein, and protein refolding, etc. (Figure 3h). Regulation of hemopoiesis and lymphocyte activation pathways were also considered. These pathways did not appear in the top ten upregulated pathways of endothelial cells between the HBV and control group (Figure 3e), indicating the limited role of cluster EC_Z1 upon CHB infection. However, cluster EC_Z2 and EC_Z3 were more likely to carry out the functional alteration of endothelial cells upon CHB infection. Results showed that upregulated DEGs of cluster EC_Z2 and EC_Z3 were both enriched in response to the lipopolysaccharide and molecules of a bacterial origin (Figure 3i,j). Moreover, NF-κB-related genes, such as *Nfkbia*, *Nfkbiz*, and *Cd36*, etc., were all shown in the network of the correspondence between the top five enriched GO terms and DEGs in EC_Z2 (Figure 4a) and EC_Z3 (Figure 4b), reconfirming the importance of the NF-κB signaling pathway in ECs upon CHB infection.

Additionally, pathways including the response to peptide and peptide hormone, the regulation of peptidase activity, and the response to virus, tumor necrosis factor and superfamily cytokine production were all upregulated in cluster EC_Z2 (Figure 3i). Apart from the pathways showed above, DEGs of cluster EC_Z3 were enriched in promoting leukocyte cell–cell adhesion and leukocyte migration, regulation of angiogenesis and vasculature development, etc. (Figure 3j). It is worth revealing the mechanisms of angiogenesis activation in pericentral ECs. The PPI network in Figure 4c, including angiogenesis-associated upregulated DEGs in pericentral ECs (Figure 4d), showed that *Hif1a*, *Itga5*, and *Shc1* may be the core genes to modulate sinusoid capillarization upon CHB infection.

In brief, the results indicated that CHB infection mainly affects midzonal and pericentral ECs with NF-κB signaling activation, instead of periportal ECs. More importantly, cell adhesion-related and capillarization-related pathways were specifically promoted in pericentral ECs.

### 2.4. Overview of Intercellular Communication between Pericentral ECs and Other Cell Populations

**Figure 5:**
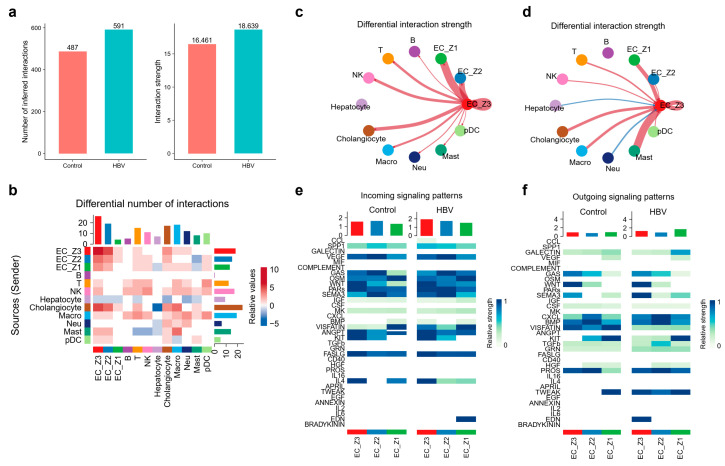
Alterations of intercellular communication patterns between healthy and HBV mice livers. (**a**) The interaction number and strength both increased in HBV mice liver. (**b**) The senders and receivers among various cell populations are shown with quantification of the differential number of interactions. (**c**,**d**) The incoming (**c**) and outgoing (**d**) intercellular interaction weight between EC_Z3 and other cell types are visualized in the circ plot. (**e**,**f**) The heatmap plot shows the relative incoming (**e**) and outgoing (**f**) signaling flow of 26 signaling pathways in ECs (including EC_Z1, EC_Z2, and EC_Z3) between healthy and HBV mice livers.

To interpret the relationship of intercellular communication and CHB progression, we investigated the changes in communication patterns between healthy and HBV mice livers by CellChat. This tool is able to quantitatively infer the intercellular communication networks, as well as predict signaling incoming and outcoming for cells. The results showed that more numerous interactions in number and strength were found in the CHB group than in control group (Figure 5a), indicating an important role of cell–cell interactions in CHB progression. Intriguingly, we showed the profile of functional changes in cellular signal networks upon CHB infection, with information about signal sources/senders and targets/receivers (Figure 5b).

Owing to the important role of pericentral ECs and its activation in cell adhesion, we considered the intricate communications and possible signaling pathways between EC_Z3 and various cell populations to reveal whether cell–cell interaction modulate the pericentral ECs function. Among all cell populations, ECs, especially EC_Z3, demonstrated stronger differential numbers of interactions than others. EC_Z3, as senders, showed high interaction with NK cells, T cells and cholangiocytes (Figure 5c). Acting as receivers, EC_Z3 showed high interaction strength with macrophages, cholangiocytes but weak interaction with others (Figure 5d), indicating that macrophages and cholangiocytes were important in changing characteristics of ECs upon HBV infection by cytokine secretion or other types of signaling. Briefly, EC_Z3 demonstrated strong intercellular communication with macrophages, cholangiocytes, T cells, and NK cells.

Apart from quantifying the overall communication number, our study detected 26 signaling pathways to decipher key signals among various cell populations, including CXCL, CCL, MIF, VISFATIN, PDGF, WNT, VEGF, IL2, and IL6, etc. To compare the incoming and outgoing signaling flow between the control and HBV group, individual signaling pathways among various cell populations were identified (Figure 5e,f). The incoming signals and outgoing signals were considered as the signals of receiving cells and secreting cells, respectively. In the HBV group, the relative incoming signaling flow of signaling VISFATIN was significantly elevated in EC_Z2 and EC_Z3, with greater changes in EC_Z3 (Figure 5e). Furthermore, signaling CCL, SPP1, and BMP were shown to mildly increase in EC_Z3. The outgoing flow of signaling CXCL, TWEAK, and EDN were elevated in EC_Z3 upon CHB infection, whereas the flow of signaling VISFATIN was decreased (Figure 5f). Therefore, we inferred that signaling VISFATIN and CXCL play a pivotal role in ECs, especially pericentral ECs, upon CHB infection.

### 2.5. Pericentral ECs Participated in Shaping Hepatic Environment through VISFATIN/Nampt and Cxcl16-Cxcl6 Signaling

**Figure 6:**
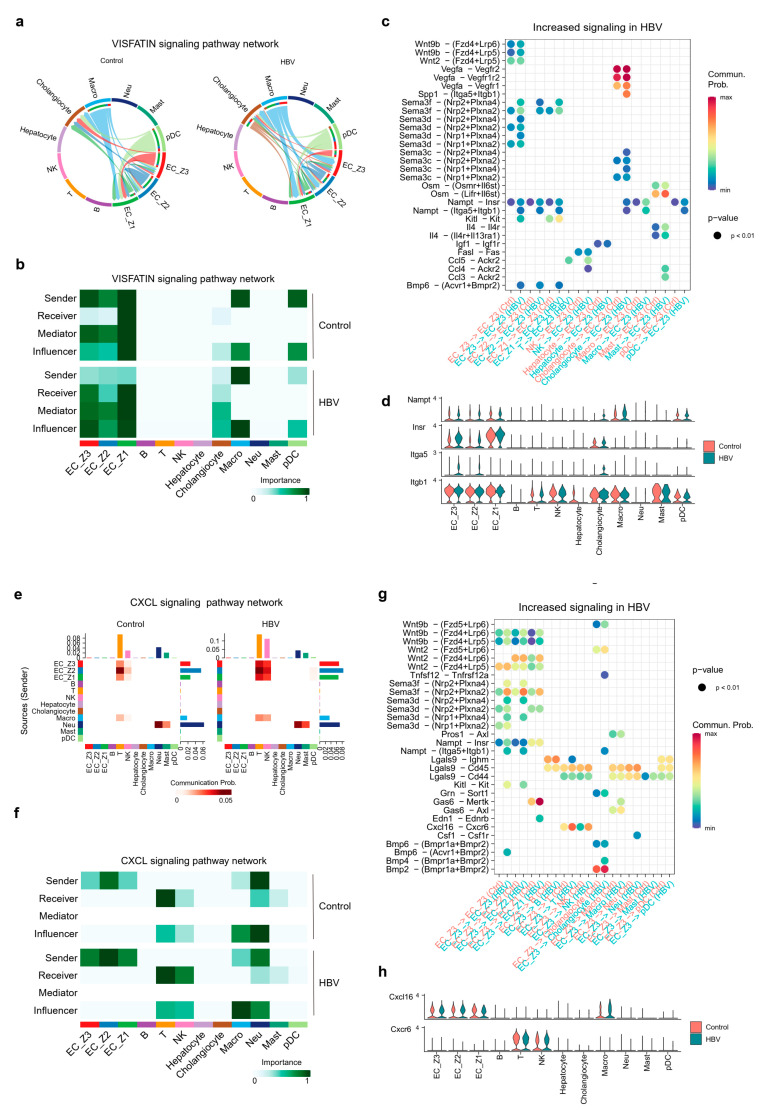
Ligand–receptor interactions analysis of signaling VISFATIN and CXCL in EC_Z3. (**a**) Circ plot shows sources and targets of signaling VISFATIN in healthy and HBV mice livers. (**b**) The score of the sender, receiver, mediator, and influencer in various cell populations indicated the role each cell type played. (**c**) Bubble plot shows the significantly differential incoming ligand–receptor interactions between EC_Z3 and other cell types. (**d**) The ligand–receptor pairs in VISFATIN were differentially expressed in source and target cells. (**e**) Hierarchy plot shows sources and targets of signaling CXCL in healthy and HBV mice livers. (**f**) The role of each cell type is visualized based on an importance score. (**g**) Bubble plot shows the significantly differential outgoing ligand–receptor interactions between EC_Z3 and other cell types. (**h**) The ligand–receptor pairs in CXCL are differentially expressed in source and target cells.

Next, the sources of secretion and individual ligand–receptor pairs in VISFATIN and CXCL signaling pathways were analyzed. On the one hand, VISFATIN signaling showed senders as downregulated and receivers as upregulated in EC_Z3 upon CHB infection, and therefore its communication flow between two groups among all cell populations were particularly visualized by the circ plot (Figure 6a). Taking the following hierarchy plot into account (Figure 6b), ECs were the principal secreting cells in healthy mice, not the receiving cells. However, ECs, especially EC_Z3 showed a high receiver and influencer score in HBV mice. Importantly, we found EC_Z3 received VISFATIN signaling which was sent from macrophages and cholangiocytes. Ligand–receptor interaction analysis demonstrated that macrophages–EC_Z3 and cholangiocytes–EC_Z3 interactions were closely related to the Nampt–Insr and Nampt–Itga5/Itgb1 pairs (Figure 6c,d). When EC_Z3 divided into pericentral LSECs and central venous ECs, we found that the Nampt–Insr and Nampt–Itga5/Itgb1 pairs were both elevated in these two subpopulations of EC_Z3 (Figure S2a,c).

Additionally, ECs transmitted signals to ECs itself via WNT, SEMA3, VISFATIN, and BMP signaling, especially the Sema3f/3d–Nrp1/2/Plxna4, Nampt–Itga5/Itgb1, Kitl–Kit, and Bmp6–Acvr1/Bmpr2 interaction pairs. NK and T cells transmitted signals to ECs via the Ccl4/Ccl5–Ackr2 pairs.

On the other hand, the communication flow of the CXCL signaling pathway were visualized by the heatmap plot (Figure 6e). In this case, ECs, with a high sender score, acted as principal secreting cells and transmitted signals to NK cells and T cells, which resulted in higher receiver and influencer scores in HBV mice than in healthy mice (Figure 6e,f). Next, we performed ligand–receptor analysis to investigate the signaling alteration in EC_Z3-T cells and EC_Z3-NK cells. We found the Cxcl16-Cxcr6 axis was elevated in both T and NK cells (Figure 6g,h), with a significantly high *Cxcl16* expression level in EC_Z3 and *Cxcr6* expression level in NK cells, reconfirming and indicating a potential role of ECs in cell adhesion and migration upon CHB infection. In addition, the Cxcl16-Cxcr6 axis played an equally important role in pericentral LSECs and central venous ECs (Figure S2b,d). Overall, we systematically constructed and compared the intercellular communication networks between healthy mice and CHB mice livers. The findings strongly suggest that the VISFATIN/Nampt signaling pathway plays pivotal parts in regulating endothelial dysfunction and vascular capillarization of ECs upon CHB infection. Subsequently, ECs showed significantly close connection with T cells and NK cells via the Cxcl16-Cxcr6 axis, which is involved in shaping the immune environment in CHB mice livers.

## 3. Discussions

In order to study the role of the hepatic microenvironment in HBV infection, we constructed the mice models with AAV8-HBV for 6 months to induce chronic inflammation in the liver. Combining subpopulation scRNA-seq analysis and intracellular communication analysis, the study comprehensively displayed the heterogeneity of ECs, revealing the mechanisms of VISFATIN/Nampt-NF-κB axis in ECs function alteration and EC-derived chemokines in the hepatic microenvironment upon CHB infection.

NF-κB signal transduction was previously reported to be activated by various stimuli, including ionizing radiation, cytokines, and neuronal injury, etc. [33]. Here, comparing with the control group, peptide-, cell adhesion-, and lipid-associated pathways of ECs in the HBV group were elevated. And we found that core DEGs such as Nfkbia, Icam1, Bcl3, Nfkbiz, Tnfaip3, Hspb1, and Cd36 involved in these pathways are all associated with NF-κB signaling, indicating the potential role of NF-κB in shaping ECs’ characteristics upon CHB infection. Nicotinamide phosphoribosyl transferase (Nampt), the rate-limiting enzyme of the NAD salvage pathway which modulates intracellular NAD concentrations [34], was reported to activate Toll-like receptor 4 (TLR4) to induce NF-κB signaling and inflammation [35]. In a previous study, VISFATIN/eNampt was also demonstrated to promote NF-κB activation as well as NLRP3 inflammasome activation through interacting with TLR4, and to impair endothelium dependent relaxations in vivo and in vitro [36]. TLR4, a pattern recognition receptor in the innate immune system, is important in generating pro-inflammatory signaling. Moreover, IκBα, encoded by *Nfkbia*, could insulate the TLR4-activated NF-κB signaling in macrophages [37]. A recent paper showed Nampt inhibition leads to the repression of NF-κB and IκBα, with increasing of TLR4 and IL-6 [38]. In our study, upregulation of Nampt expression levels in macrophages and cholangiocytes and NF-κB activation in ECs were both found in CHB mice livers, indicating the role of VISFATIN/Nampt- NF-κB axis in endothelial dysfunction and downstream hepatic inflammation. Moreover, after unraveling the heterogeneity of three EC subpopulations, we disclosed that midzonal ECs and pericentral ECs may be more sensitive to HBV infection in livers, for the VISFATIN/Nampt- NF-κB axis was mainly altered in these two subpopulations.

Capillarization is a typical phenotype in LSECs during liver fibrotic progression [23], which was specifically found in pericentral ECs upon CHB infection based on the functional enrichment data and PPI network, giving us the potential mechanisms of capillarization with hub genes like *Hif1a*, *Itga5*, and *Shc1*, etc. Interestingly, pericentral ECs showed increasing communication with macrophages and cholangiocytes via the VISFATIN pathway through the Nampt–Insr and Nampt–Itga5/Itgb1 pairs upon CHB infection. Both *Insr* and *Itga5* are correlated with capillarization and vasculature development [39,40]. Previous work showed that endothelial insulin receptors (Insr) are enriched in tip ECs, leading to VEGF signaling and VEGFR2 internalization, which promotes sprouting angiogenesis and EC migration [40]. Integrin α5 (Itga5) was also reported to promote angiogenesis through VEGFA in cervical cancer [39]. Our study found both the Insr and Itga5 expression levels were significantly upregulated in pericentral ECs, instead of midzonal ECs or periportal ECs, indicating angiogenic sprouting associated with the Insr/Itga5-VEGF axis occurs in pericentral ECs upon CHB infection.

Thus, elevated macrophages–ECs and cholangioctes–ECs interactions via Nampt-Insr and Nampt-Itga5/Itgb1 were found in CHB mice livers, indicating the role of VISFATIN/Nampt- NF-κB axis in endothelial dysfunction and the Insr/Itga5-VEGF axis in angiogenesis.

Apart from participating in hepatic inflammation, ECs have an important role in promoting the recruitment and retention of immune cells in liver [41,42] by secreting CXCL9, CXCL11, and CXCL16, etc. However, the role of ECs in modulating the liver microenvironment during CHB infection remains unclear. In our study, intracellular communications between pericentral ECs and NK/T cells were increased upon CHB infection via the Cxcl16-Cxcr6 axis. CXCR6 is a chemokine receptor for CXCL16, as well as an important marker for T cells in antitumor immunity [43]. It was previously reported that CXCR3^+^CXCR6^+^ γδT cells, a type of liver-resident T cell, are protective against acute HBV infection [41]. Thus, our study investigated the signaling sending by ECs, indicating the role of ECs in recruiting NK cells and T cells. Additionally, HBV-specific CD4+ T cells are thought to be exhausted due to the reduced cytokine secretion and elevated expression of PD-1 and CTLA-4. The impaired T cell responses induced by CHB are also an important reason for the chronicity of HBV infection. The study showed that HBsAg and HBeAg inhibited the activation, cytokine production, and cytotoxic granule release in NK cells through STAT- and MAPK-related pathways, which might contribute to HBV persistence. In brief, endothelial cells could not only induce hepatic inflammation itself, but contribute to the chronicity of HBV infection by recruitment of impaired T cells or NK cells.

From a technical perspective, we used known gene markers which related to spatial distribution as described in the previous papers to annotate endothelial cells [22,24,29,30]. And those markers were performed for the first time to study HBV infection. Unsurprisingly, the importance of endothelial cells has been found in various liver pathologies, such as hepatic fibrosis [12], alcohol-associated liver disease [44], non-alcoholic steatohepatitis (NASH), viral hepatitis [45,46,47], and liver transplant rejection, etc. [48]. Endothelial cells play multiple roles in HBV infection. For example, a study showed that LSECs-secreted epidermal growth factor (EGF) modulates HBV infection in a dose-dependent manner [46]. Endothelial progenitor cells (EPCs) could serve as a virus carrier and be effectively infected by the uptake of HBV in vitro [47]. Another study indicated that the antigen-presenting ability of LSECs inducing by a NOD1 ligand (diaminopimelic acid [DAP]) was critical in enhancing T cell responses and inhibited HBV replication [15]. Further, HBeAg could induce LSEC maturation and trigger intrahepatic HBV-specific T cell activation in a TNF-α dependent manner [16]. In the study, all cell types in hepatic microenvironment were considered as a whole, comprehensively demonstrating the possible connection of endothelial cells and other cells in the hepatic microenvironment, as well as providing clues for subsequent research.

Collectively, the study described a transcriptional profile of ECs, which reveals the complex mechanisms by HBV to orchestrate the functional alteration of ECs and its subpopulations in hepatopathologies. ECs could not only act as recipients to sense the macrophage- and cholangiocyte-derived VISFATIN/Nampt signaling and affect downstream hepatic inflammation, but also senders that are secreting Cxcl16 to recruit Cxcr6^+^ NK cells and T cells, which imply the feasibility of modulating ECs dysfunction, vascular capillarization, and cell–cell interaction via potential targets in combating the progression of CHB pathologies.

## 4. Materials and Methods

### 4.1. Mice

Wide-type male C57BL/6J (6 weeks of age) mice were purchased from Beijing Vital River Laboratory Animal Technology Co., Ltd. (Vital River Laboratory Animal Technology, Beijing, China) with license No. SCXK-(jing) 2016-0006. All animals were housed in pathogen-free conditions and a temperature-controlled room with a 12-h light/dark cycle, adequate water, and food. Mice were infected with HBV and grew to 6 months after infection. Animal procedures were according to guidelines for the care and use of laboratory animals prepared by the animal welfare and ethics committee of the Animal Facility of AMMS, Beijing, China (Approval No. IACUC-IME-2023-036).

### 4.2. AAV-HBV Mice Construction

AAV8-1.3HBV (D genotype HBV ayw genome) was purchased from Guangzhou PackGene Biotech Institute (PackGene Biotech Institute, Guangzhou, China). Intravenous injection of 1.0E11GE AAV8-HBV or AAV8-GFP was performed on wild-type 6-week-old male C57BL/6J to construct the HBV mice model or control mice model, respectively. Serum HBsAg, HBeAg, HBV DNA, and A/G levels were regularly detected after viral injection. HBsAg and HBeAg were detected based on the time-resolved fluoroimmunoassay (TRFIA) with test kits purchased from Guangzhou DARUI Biotechnology Co., Ltd. (DARUI Biotechnology, Guangzhou, China). HBV DNA levels were measured by Real-time qPCR with test kits purchased from Hunan Sansure Biotech Inc (Sansure Biotech, Changsha, China). A/G levels were measured with the automatic biochemical analyzer (MNCHIP, Tianjin, China). Student’s *t*-test was used to calculate the statistical significance.

### 4.3. Histological Staining

Liver tissues were treated with 4% paraformaldehyde for about 48 h, followed by embedding and sectioning (5 μm). Standard hematoxylin and eosin (H&E) staining were performed for evaluating the inflammation and pathological condition of mice livers. Moreover, immunohistochemistry on liver sections was performed to detect HBsAg, a marker protein of HBV infection. In brief, sections were treated with de-paraffinization and rehydration, followed by antigen retrieval with sodium citrate (Zsbio, Beijing, China). After 3% H_2_O_2_ (Zsbio, Beijing, China) and serum blocking, liver sections were stained in primary HBsAg antibody (1:400, ab68520, Abcam, Cambridge, UK) overnight at 4 °C. After primary antibody incubation, sections were treated with secondary antibody (Zsbio, Beijing, China) for 20 min at room temperature. Then, the DAB method was used for visualizing the positive expression of target markers and cell nucleus that were mounted with hematoxylin staining.

### 4.4. Preparation of Single-Cell Suspensions of Mice Livers

Hepatocytes and liver nonparenchymal cells were extracted from liver tissue samples performed by the mouse liver dissociation kit (Miltenyi Biotec, Bergisch Gladbach, Germany) for 40 min according to the manufacturer’s protocol. After dissociation with heaters, cells were filtered by a 70 μm smartStrainer with a total of two or three times of washing; this depended on whether cell viability is necessary for us to perform cell debris removal with Debris Removal Solution (Miltenyi Biotec, Bergisch Gladbach, Germany). Then, lysing of erythrocytes with RBC lysis buffer (Solarbio, Beijing, China) was indispensable.

### 4.5. ScRNA-Seq Library Construction and Sequencing

Cells were isolated from liver tissues and acted as the input of the chromium system (10X Genomics, Pleasanton, CA, USA) on the single cell chip G. Single-cell gel beads were generated and the library was constructed following the manufacturer’s guidelines. Next, double-end sequencing was performed on the MGI-2000H (MGI Tech Co., Ltd., Shenzhen, China) platform using PE150 with a 28 bp length of Read1 and 91 bp length of Read2.

### 4.6. Quality Control (QC) and Cell Type Annotation

After sequencing, the count matrix was constructed from data mapping with the mouse genome by using the Cell Ranger software (version 6.1.2) from 10X Genomics and integrated by using the Seurat (version 4.3.0) R package. After building the Seurat object, we performed a stricter QC to filter high quality cells according to the following criteria: (1) number of genes expressed >500, (2) the number of UMI counts >800, and (3) the percentage of mitochondrial read count <10%. Additionally, we discarded cells which were out of the limit of the mean value ± 2-fold of standard deviations (SD) based on a Guassian distribution. After QC for each sample, single cells of a high quality were included.

A function of Seurat called *FindVariableFeatures* was used to identify the top 3000 highly variable features. Then the expression values of those features were scaled before carrying out dimensional reduction and principal component analysis (PCA). Top 30 PCs were measured to identify 36 clusters and visualize the TSNE embedding with a 1.0 resolution. According to known marker genes previously reported, 10 cell types were annotated for downstream analysis. The marker genes were listed as follows: T cells (marked with *Cd3d*, *Cd3e*); B cells (marked with *Cd79a* and *Cd79b*); natural killer (NK) cells (marked with *Il2rb*, *Nkg7*, *Klrb1c* and *Klrk1*); ECs (marked with *Pecam1*, *Nrp1*, *Kdr*, and *Oit3*); hepatocyte (marked with *Alb*, *Apoa1*, *Fgb*, *Gc*, *Ahsg*, and *Car3*); cholangiocyte (marked with *Sox9* and *Epcam*); macrophage (marked with *Lyz2*, *Itgam*, and *Mpeg1*); neutrophils (marked with *S100a8* and *S100a9*); mast cell (marked with *Mcpt8* and *Ms4a2*); and dendritic cells (DCs) (marked with *Siglech*).

### 4.7. Identification of DEGs and Gene Ontology (GO) Enrichment Analysis

A function of Seurat called *FindMarker* was used to identify the differentially expressed genes (DEGs) of each cell type between the control group and HBV group according to the following threshold criteria: (1) average log2(fold change) ≥ 0.5, (2) the adjust *p* value < 0.05. We used the Volcano plot or Manhattan plot to visualize the DEGs. After DEGs filtering, Gene Ontology (GO) enrichment analysis with the *org.Mm.eg.db* database and *enrichGO* function by the ClusterProfiler [49] software (version 4.6.2) was utilized to reveal the main functional alteration of the cell type. The Benjamini–Hochberg (BH) method was used for the multiple test adjustments. Further, ggplot2 (version 3.4.2) was used for visualization.

### 4.8. Protein–Protein Interaction (PPI) Network Construction

We identified the DEGs of the ECs subgroup between the control and HBV mice and selected those DEGs of pathways of interests to construct biologically functional networks. The STRING database was used to analyze and visualize PPI networks, which served genes as nodes and inferred the most important genes among massive information. As for the active interaction sources, the inclusion criteria were as follows: (1) text mining, neighborhood, gene fusion, co-occurrence, experiments, databases, and co-expression information were considered; (2) the minimum required interaction score was set to 0.400. Finally, the most important genes were in the center of the network, which were those with the most protein–protein associations edges.

### 4.9. Cellular Communication Networks Analysis

The CellChat [50] R package (https://github.com/sqjin/CellChat, accessed on 1 July 2022) was used to calculate, analyze, and infer intercellular communication from single-cell transcriptional data. In the study, hepatocytes, cholangiocytes, ECs (including periportal ECs, midzonal ECs, and pericentral ECs), and immune cells (including T cells, B cells, NK cells, macrophages, neutrophils, and dendritic cells) were extracted for intercellular communication analysis. We set the expression matrix of each cell type as the input for CellChat to reveal significantly overexpressed ligand–receptor pairs. Statistical tests were used to find significant interactions with *p* value < 0.05. Moreover, we used CellChat to visualize the ligand–receptor pair between endothelial cells and other cells. Firstly, we considered other cells as source cells and pericentral ECs as target cells to infer a signaling pathway associated with ECs’ functional alteration. Secondly, we considered pericentral ECs as the source cells and others as targets to infer the downstream influence. The heatmap plot or circ plot was performed to visualize the communication probabilities or significant signaling pathways among the source and target cells.

## Data Availability

The data that support the findings of this study are available from the corresponding authors on request.

## Supplementary Materials

The following supporting information can be downloaded at: https://www.mdpi.com/article/10.3390/ijms25137016/s1.
