# Supplementary material for: Single-Cell RNA Sequencing Reveals the Spatial Heterogeneity and Functional Alteration of Endothelial Cells in Chronic Hepatitis B Infection
Source: Int J Mol Sci. 2024 Jun 27;25(13):7016. doi: 10.3390/ijms25137016 (PMC11241719; doi:10.3390/ijms25137016)
Supplement: Supplementary file 1 [file ijms-25-07016-s001.zip › ijms-3023007-supplementary.pdf]

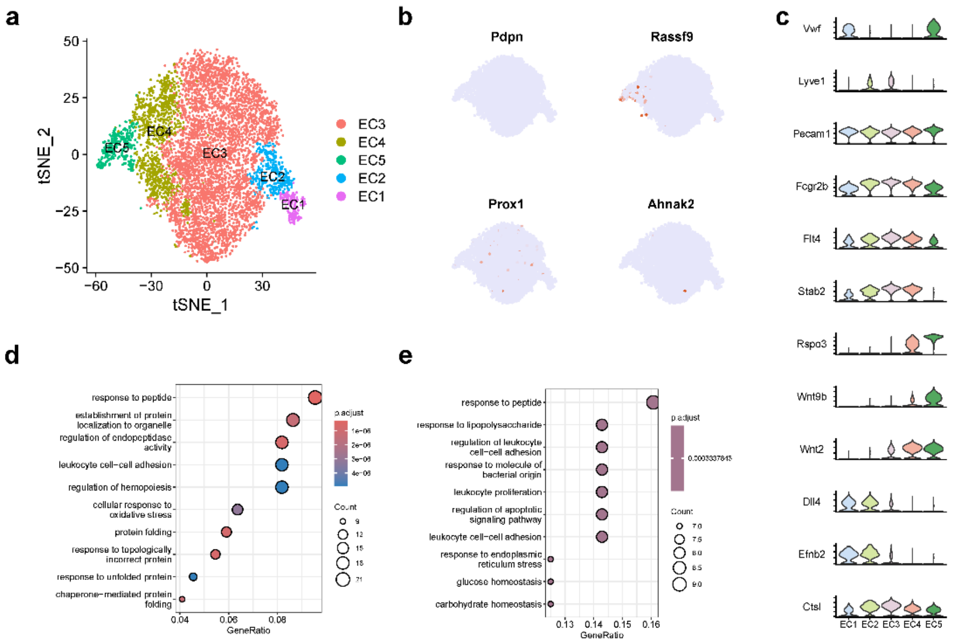

Supplementary Figure S1. Spatial heterogeneity of ECs. (a) t-SNE plot showed 5 clusters of ECs. (b) t-SNE plot showed the representative lymphatic ECs marker gene expression. (c) Violin plot showed the representative ECs marker gene expression. (d-e) GO enrichment analysis showed the up-regulated pathways in EC4 (d) and EC5 (e).

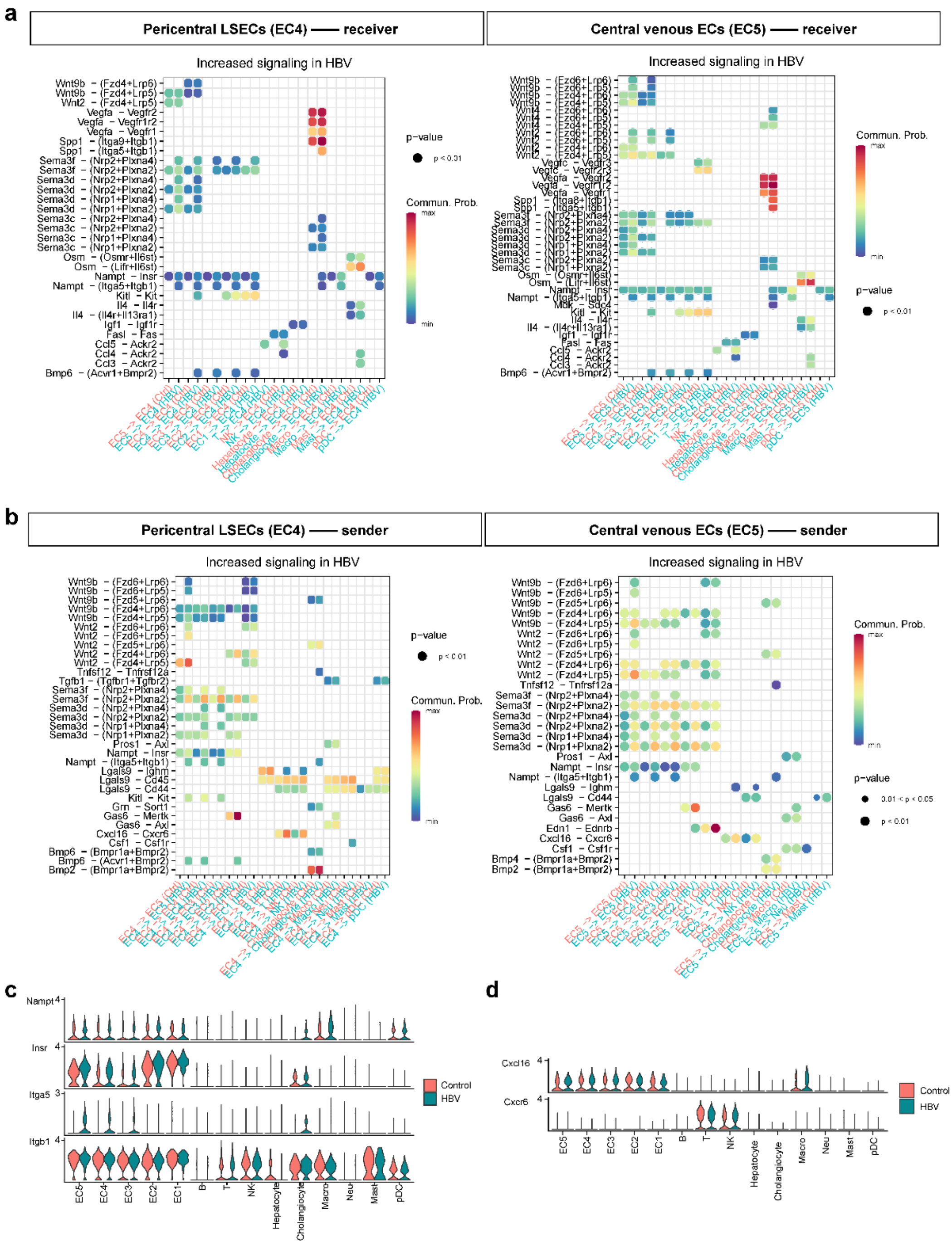

Supplementary Figure S2. Ligand-receptor interactions analysis in pericentral LSECs and central venous ECs. (a) Bubble plot showed the significantly differential incoming ligand-receptor interactions of pericentral LSECs (left) and central venous ECs (right). (b) Bubble plot showed the significantly differential outcoming ligand-receptor interactions of pericentral LSECs (left) and central venous ECs (right). (c) The ligand-receptor pairs in VISFATIN were differentially expressed in source and target cells. (d) The ligand-receptor pairs in CXCL were differentially expressed in source and target cells.
